# Surgery May Be a Major Contributor for Postoperative Delirium in Patients With Elective Thoracic Aortic Aneurysm Procedures

**DOI:** 10.1111/cns.70509

**Published:** 2025-07-09

**Authors:** Lucas G. Fernandez, Weiran Shan, Abdullah S. Terkawi, Sandeep Yerra, Zhiyi Zuo

**Affiliations:** ^1^ Department of Anesthesiology University of Virginia Charlottesville Virginia USA

**Keywords:** anesthesia, body temperature, inflammation, postoperative delirium, surgical trauma

## Abstract

**Aims:**

Postoperative delirium (POD) is relatively common and is associated with poor outcomes. Age is a risk factor for POD. This single‐center observational study is designed to determine whether surgery is a major contributor to the development of POD and inflammatory response.

**Methods:**

Patients with elective procedures to repair thoracic aortic aneurysm were recruited to the study. Confusion Assessment Method was used to assess POD. Their blood and cerebrospinal fluids were harvested for analysis of inflammatory and neuronal injury indicators.

**Results:**

A total of 67 patients were included in this study: 32 had stent placement under general anesthesia and 35 had open surgery to repair the aneurysm. No patients in the stent placement group had POD, but 9 patients in the open surgery group had POD (25.7%). Patients with POD had a lower body temperature at the end of surgery than patients without POD [36.3°C (35.7°C–36.6°C) vs. 36.7°C (36.3°C–37.0°C), *p* = 0.046]. This parameter was identified as a risk factor for POD. Patients in the open surgery group had increased interleukin 1β and neurofilament light chain in the blood. However, there was no change in these biomarkers in the cerebrospinal fluids at 10 and 24 h after surgery.

**Conclusion:**

Our results suggest that surgery is a major contributor to POD, inflammatory response, and neuronal injury. Low body temperature at the end of surgery is a potential risk factor for POD in patients with open repair for thoracic aortic aneurysm.

## Graphical abstract

Diagram of findings: Surgical trauma is a major contributor to postoperative delirium, inflammation, and neuronal injury. Low body temperature at the end of surgery may be a risk factor for postoperative delirium in patients with open surgery repair for thoracic aortic aneurysm.

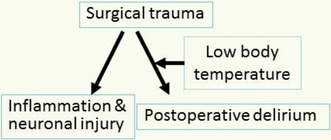

## 1 Introduction

More than 50 million people each year in the United States have surgery and about 25% to 35% of them are older than 65 years when they have surgeries [1]. Postoperative delirium (POD) affects 15% to 60% of the elderly patients after surgery [2, 3]. POD is a type of brain dysfunction characterized by fluctuating disturbances in attention, cognition, and awareness after surgery [4, 5]. Patients with POD have a longer hospital stay and intensive care unit (ICU) stay. POD is also associated with a higher mortality rate [4, 5, 6]. Thus, POD is a significant clinical issue. Multiple factors, such as aging and preoperative cognitive impairment, have been identified as risk factors for POD [4, 7, 8]. However, many of these factors are not modifiable during the perioperative period.

Our previous animal study has shown that surgical trauma is the major contributor to the development of postoperative cognitive dysfunction (POCD) [9], a syndrome that is related to POD [10]. It has been proposed that surgical trauma is a contributing factor for POD [11]. However, clear evidence to support this possibility is limited. General anesthesia may be a factor for POD [12, 13]. However, patients with surgery under neuraxial block also develop POD [12, 13].

One of the underlying mechanisms for POD is neuroinflammation [14, 15]. It is known that systemic inflammation can induce neuroinflammation [16]. This study compared the POD incidence and levels of inflammatory cytokine and neuronal injury biomarker in the blood and cerebrospinal fluids (CSF) of patients undergoing elective thoracic aortic aneurysm surgery repair or stent placement to determine whether surgical trauma is a major factor for the development of POD, neuroinflammation, and systemic inflammation.

## 2 Materials and Methods

No artificial intelligence tools were used in the writing of the manuscript, production of images, or the collection and analysis of data.

### 2.1 Selection of Subjects

Patients undergoing elective thoracic aortic aneurysm surgery or stent placement at the University of Virginia Medical Center between 2012 and 2020 were recruited for this study. One additional inclusion criterion was that the placement of a lumbar drain before the surgery or procedure was a planned event.

Patients were excluded if they had: (a) any known central nervous system (CNS) diseases, (b) peripheral muscle weakness or sensory impairment that would confuse the diagnosis of spinal cord injury after the anesthesia or surgery, (c) steroid therapy within the past 6 months or during the perioperative period via any routes including oral, intravenous, or inhalational routes (recruitment for this study had to be stopped in 2020 because per new surgical protocol all patients for thoracic aortic aneurysm procedures would receive steroids during the perioperative period after 2020), and (d) known immunological diseases.

Informed consent was obtained from all participants before the start of the procedure, including blood and CSF collection, and clinical data collection. All procedures were approved by the University of Virginia Institutional Review Board (protocol number: 15903). Online registration was not performed because this study was an observation study.

### 2.2 CSF and Blood Collection

After the lumbar drain was placed, we collected 3 mL CSF from the drainage tube at the following time points: immediately before surgery, at the conclusion of surgery, and 10, 24, and 48 h after surgery or procedure, if lumbar drain remained in place. CSF was stored at −80°C.

Blood samples (3 mL) were also collected through an existing arterial or intravenous line at pre‐surgery, the end of surgery, and 24 h post‐surgery. Blood was processed immediately to prepare plasma that was stored at −80°C for future analysis.

It was very unfortunate that we only had the plasma samples of the last 10 participants. Among them, 5 participants were in the stent placement group and the other 5 participants were in the open surgery group (one participant in each group missed the sample at 24 h after surgery). The plasma samples of other participants were accidentally thrown away by a colleague because the samples looked old in dates to the person. Similarly, we had CSF samples of the last 34 participants. Most participants did not have the CSF sample at 48 h after the surgery because the lumbar drain was removed before the time point. The decision was made to use the CSF samples of the same last 10 participants plus 3 more participants from each group. The selection of these 3 additional participants was that they needed to have one CSF sample that was collected at least 10 h after the surgery because of the consideration that it would take some time for the brain biochemical changes to occur after surgery.

### 2.3 Collection of Clinical Data

Clinical data collected included: (1) demographics and medical history: age, gender, history of smoking, alcohol use, and non‐steroidal anti‐inflammatory drugs (NSAID) use, diabetes, and American Society of Anesthesiologists (ASA) physical status, and (2) surgical/anesthesia data: length of anesthesia and surgery or procedure, major anesthetics, use of cardiopulmonary bypass, and temperature at the end of surgery.

### 2.4 Assessment of Postoperative Delirium

The diagnosis of delirium was based on Confusion Assessment Method (CAM) [17] or CAM for the ICU (CAM‐ICU) [18]. Medical records were reviewed to find documented positive CAM assessments performed by nurses or other health care providers, at least twice a day, in all patients during the first seven days after surgery or until discharge. We recorded the post‐operative day(s) and time(s) when the positive CAM results were obtained.

### 2.5 Analysis of the CSF and Plasma

Interleukin (IL)‐1β concentrations in the plasma and CSF were determined by human IL‐1β/IL‐1F2 ELISA Kit (catalogue number: DLB50, R&D System, Minneapolis, MN). Many cytokines, including IL‐1β and IL‐6, had been reported to increase after surgery, and the increase was associated with the occurrence of POD [19, 20]. IL‐1β was chosen to be measured because it can increase the expression and release of other cytokines, such as IL‐6, to enhance inflammatory responses [21]. Neurofilament light chain (NFL) levels in the plasma and CSF were determined by human NFL/NEFL (Sandwich ELISA) ELISA Kit (catalogue number: LS‐F6701, Lifespan Biociences, Newark, CA). Corticosterone and adrenaline concentrations in the plasma were determined by corticosterone ELISA kit (catalogue number: ADI‐900‐097, Enzo Life Sciences, Farmingdale, NY) and adrenaline/epinephrine (Competitive EIA) ELISA Kit (catalogue number: LS‐F25682, Lifespan Biociences), respectively. These assays were performed according to the manufacturers' instructions and as we did previously [22]. The levels of IL‐1β, NFL, corticosterone, and adrenaline at late time points were normalized by the corresponding pre‐surgery concentrations to reduce the effects of missing samples/data at a late time point in some participants on the mean and SD of the group.

### 2.6 Statistical Analysis

This is an observational study with the goal to recruit as many qualified patients as possible. The specific target number of participants was not decided prior to the study, but the inclusion and exclusion criteria were set before the study. The study was stopped due to the introduction of a new surgical practice that was the use of steroids during the perioperative period for all thoracic aortic aneurysm procedures. Data of all patients who consented to the study were included, except for the blood and CSF results due to the loss of the samples. *Post hoc* power analysis was performed to determine the likelihood of detecting a difference in the risk for POD between patients with open surgery and stent placement.

Parametric data in normal distribution were presented as mean ± SD. Non‐parametric data or parametric data in non‐normal distribution were presented in median and interquartile range, or percentage. The determination of normality of the data was performed by the Kolmogorov–Smirnov test. Data of CSF and plasma from various time points were analyzed by a two‐way repeated measures analysis of variance followed by the Tukey test. The other data were tested by t‐test, Mann–Whitney rank sum, Fisher exact, or Chi‐square tests as appropriate. Multivariate analyses were performed to identify potential risk factors for POD in patients with open surgery for thoracic aortic aneurysm surgery. Variables that are known to be associated with POD or had a *P* value of < 0.05 in the univariate analysis were selected for the multivariate analysis. Differences were considered significant at *p < 0.05*. All statistical analyses were performed with SigmaStat (Systat Software Inc., Point Richmond, CA).

## 3 Results

**FIGURE 1:**
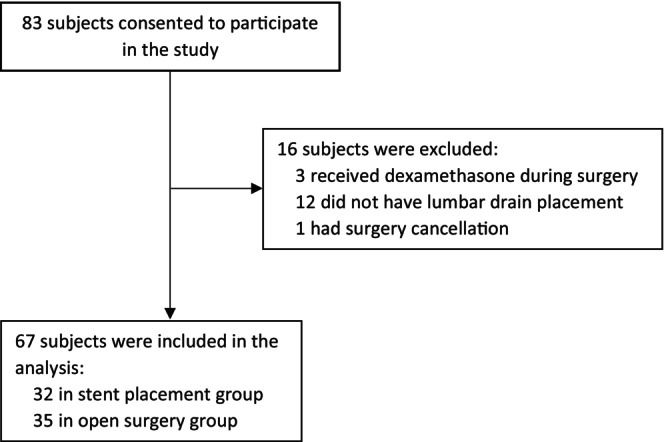
Flow diagram of recruitment.

A total of 67 participants were in our study. Among them, 32 had a stent placement procedure and 35 had open surgery (Figure 1). All participants had their procedure or surgery under general anesthesia. Their general characteristics were presented in Table 1. Interestingly, patients with open surgery had a higher ASA physical status classification than patients with stent placement, suggesting that patients with open surgery are sicker. As expected, none of the patients in the stent placement group had cardiopulmonary bypass (CPB) and the majority of patients with open surgery had CPB. Patients with open surgery had a much longer procedure time and anesthesia time. They stayed in the ICU and hospital longer than those patients with stent placement (Table 1).

**TABLE 1:** General characteristics of patients.

	Stent placement *N* = 32	Open surgery *N* = 35	*p*
Age (year), median (25th—75th percentile)	67 (60–72)	63 (52–73)	0.139
Sex			
Male, *n* (%)	20 (62.5%)	22 (62.9%)	1.000
Female, *n* (%)	12 (37.5%)	13 (37.1%)	
ASA physical status level			0.036
I and II, *n* (%)	2 (6.3%)	0 (0%)	
III, *n* (%)	19 (59.4%)	13 (37.1%)	
IV, *n* (%)	11 (34.3%)	22 (62.9%)	
Diabetes, *n* (%)	7 (21.9%)	5 (14.3%)	0.529
Chronic kidney disease, *n* (%)	4 (12.5%)	5 (14.3%)	1.000
Smoking history, *n* (%)	24 (75.0%)	26 (74.3%)	1.000
History of alcohol use, *n* (%)	8 (25.0%)	16 (45.7%)	0.125
History of NSAID use, *n* (%)	26 (81.3%)	30 (85.7%)	0.746
Duration of procedure/surgery (min), mean (SD)	135 (62)	443 (83)	< 0.001
Duration of anesthesia (min), mean (SD)	256 (80)	586 (90)	< 0.001
Main anesthetics			0.143
Propofol, *n* (%)	0 (0%)	3 (8.6%)	
Sevoflurane, *n* (%)	31 (96.9%)	32 (91.4%)	
Desflurane, *n* (%)	1 (3.1%)	0 (0%)	
Use of CPB, *n* (%)	0 (0%)	33 (94.3%)	< 0.001
Planned hypothermia, *n* (%)	3 (9.4%)	26 (74.3%)	< 0.001
Temperature at the end of surgery (°C), mean (SD)	36.0 (0.7)	36.5 (0.8)	0.005
Duration of ICU stay (h), median (25th—75th percentile)	52 (30–72)	93 (63–136)	0.002
Duration of hospitalization (day), median (25th—75th percentile)	5 (4–6)	8 (7–13)	< 0.001

Abbreviations: ASA, American Society of Anesthesiologists; CPB, cardiopulmonary bypass; ICU, intensive care unit; NSAID, nonsteroidal anti‐inflammatory drug.

Among the 9 participants who had POD, 5 participants had POD on postoperative day 1 and one participant each had POD on postoperative day 0, 2, 3, and 6. Two participants who had POD on postoperative day 1 had POD again on postoperative days 2 and 3, respectively. Remarkably, all patients with POD were in the open surgery group (Table 2). The incidence of POD within 2 days after surgery and during the whole hospitalization period was 20% and 25.7%, respectively. Patients with open surgery were more likely to develop POD than patients with stent placement. The *post hoc* calculated power to detect this difference (*α* = 0.05) in our study was 0.628 and 0.800 for POD within 2 days after surgery and during the whole hospitalization period. One participant in the stent placement group had some delirium features (short form CAM score: 2) within 30 min after the end of anesthesia and did not have the delirium presentation anymore during the rest of the hospitalization. This participant may suffer from emergence delirium [23], which may be related to an inadequate wakening process from anesthesia [23]. In addition, many studies on POD start POD evaluation from postoperative day 1 [24, 25, 26]. Thus, this participant was not classified as a case with POD in our study. Another participant from open surgery had a full delirium presentation 3.3 h after the end of anesthesia This time is out of the usual timeframe of emergence delirium/agitation (up to 45 min after anesthesia) [23] and was classified as a case with POD.

**TABLE 2:** POD incidence.

	Stent placement *N* = 32	Open surgery *N* = 35	*P*	Power (*post hoc*)
Within 2 days after surgery				
POD, *n* (%)	0 (0%)	7 (20.0%)	0.012	0.628
Non‐POD, *n* (%)	32 (100%)	28 (80.0%)		
During hospitalization stay				
POD, *n* (%)	0 (0%)	9 (25.7%)	0.002	0.800
Non‐POD, *n* (%)	32 (100%)	26 (74.3%)		

To determine the factors that were associated with POD, univariate analysis was performed by using the data of patients with open surgery. Patients with POD had a low body temperature at the end of surgery (Table 3). To further determine the role of low body temperature in POD, multivariate analysis was performed. The variables that were used in the model included age, a well‐recognized risk factor for POD [4, 7, 8], and those characteristics whose comparison between stent placement and open surgery groups had a *P* value of < 0.05 in Table 1. We used parameters with a *P* value of < 0.05 in Table 1 but not in Table 3 because there is only one parameter with a *P* value of < 0.05 in Table 3 (low body temperature at the end of surgery) and all POD patients were in the open surgery group (and thus, the differences between open surgery and stent placement groups may be risk factors). Our results showed that only low body temperature at the end of surgery is an independent risk factor for POD in patients with open surgery (Table 4).

**TABLE 3:** Univariate analysis comparing patients with and without POD after open surgery.

	Patients with POD *N* = 9	Patients without POD *N* = 26	*p*
Age (year), mean (SD)	58.3 (19.9)	60.1 (15.4)	0.788
Sex			1.000
Male, *n* (%)	6 (66.7%)	16 (61.5%)	
Female, *n* (%)	3 (33.3%)	10 (38.5%)	
ASA physical status level			0.185
I and II, *n* (%)	0 (0%)	0 (0%)	
III, *n* (%)	5 (55.6%)	8 (30.8%)	
IV, *n* (%)	4 (44.4%)	18 (69.2%)	
Diabetes, *n* (%)	2 (22.2%)	3 (11.5%)	0.586
Chronic kidney disease, *n* (%)	1 (11.1%)	4 (15.4%)	1.000
Smoking history, *n* (%)	6 (66.7%)	20 (76.9%)	0.665
History of alcohol use, *n* (%)	5 (55.6%)	11 (42.3%)	1.000
History of NSAID use, *n* (%)	7 (77.8%)	23 (88.5%)	0.586
Duration of procedure/surgery (min), mean (SD)	401 (78)	457 (81)	0.083
Duration of anesthesia (min), mean (SD)	550 (87)	598 (89)	0.174
Main anesthetics			0.287
Propofol, *n* (%)	0 (0%)	3 (11.5%)	
Sevoflurane, *n* (%)	9 (100%)	23 (88.5%)	
Desflurane, *n* (%)	0 (0%)	0 (0%)	
Use of CPB, *n* (%)	8 (88.9%)	25 (96.1%)	1.000
Planned hypothermia, *n* (%)	5 (55.6%)	21 (80.8%)	0.192
Temperature at the end of surgery (°C), median (25th—75th percentile)	36.3 (35.7–36.6)	36.7 (36.3–37.0)	0.046
Duration of ICU stay (h), median (25th—75th percentile)	94 (49–350)	91 (67–120)	0.850
Duration of hospitalization (day), median (25th—75th percentile)	8.0 (6.8–21.5)	8.5 (7.0–13.0)	0.909

Abbreviations: ASA, American Society of Anesthesiologists; CPB, cardiopulmonary bypass; ICU, intensive care unit; NSAID, nonsteroidal anti‐inflammatory drug.

**TABLE 4:** Multivariate analysis comparing patients with and without POD after open surgery.

	Odds ratio (95% CI)	*p*
Age	1.008 (0.920–1.104)	0.871
ASA physical status level	0.485 (0.056–4.222)	0.512
Duration of procedure/surgery	0.974 (0.925–1.024)	0.303
Duration of anesthesia	1.019 (0.974–1.065)	0.410
Use of CPB	20.944 (0.151–2906.408)	0.227
Planned hypothermia	0.177 (0.0107–2.951)	0.228
Temperature at the end of surgery	0.162 (0.028–0.955)	0.044

Abbreviations: ASA, American Society of Anesthesiologists; CI, confidential interval; CPB, cardiopulmonary bypass.

**FIGURE 2:**
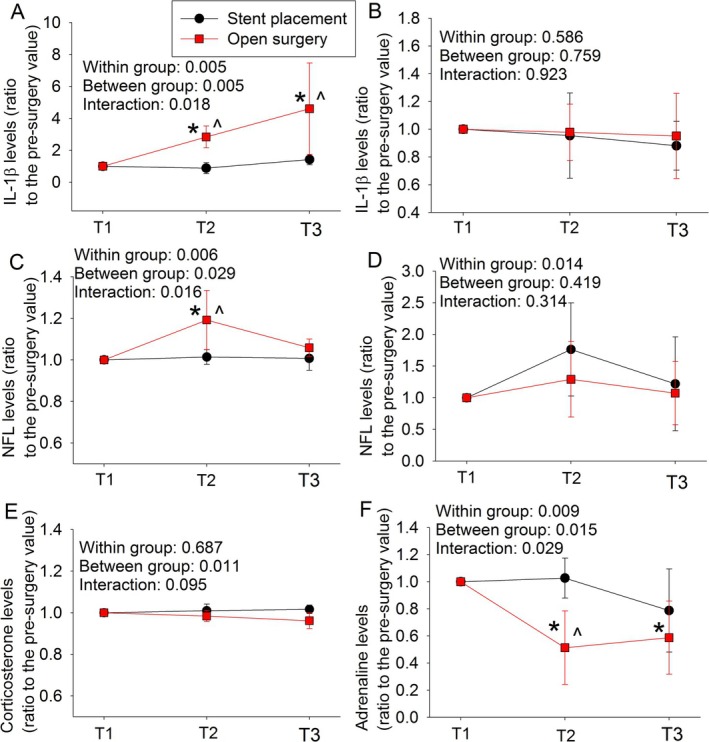
Comparison of cytokine, neuronal injury biomarker, and stress biomarkers between patients with open surgery and stent placement. Panels A, C, E, and F are plasma results. Panels B and D are CSF results. The *p* values presented in each panel are the results of two‐way repeated measures ANOVA with time and procedure types as two factors in the analysis. Results are mean ± SD (*n* = 4–5 for plasma samples and = 6–8 for CSF samples). T1, T2, and T3 are before anesthesia and surgery, at the end of surgery/procedure, and 24 h after surgery/procedure for plasma samples, and before anesthesia and surgery, 10 h and 24 h after surgery/procedure for CSF samples. *p < 0.05 compared with the pre‐surgery/pre‐procedure values. ^*p* < 0.05 compared with the corresponding values of patients with stent placement.

The levels of IL‐1β in the plasma were increased at the end of the surgery and 24 h after the surgery in patients with open surgery but were not changed in patients with stent placement (Figure 2A), suggesting an inflammatory response in the patients with open surgery. However, the levels of IL‐1β in the CSF of patients with open surgery and stent placement were not changed at 10 and 24 h after the surgery/procedure compared with the pre‐surgery/procedure levels (Figure 2B). The levels of NFL in the plasma of patients with open surgery were increased at the end of surgery (Figure 2C), indicating neuronal injury in these patients. The levels of NFL in the CSF were not changed at 10 and 24 h after surgery/procedure (Figure 2D). Finally, the levels of plasma corticosterone were not changed for both patients with open surgery or stent placement (Figure 2E) but the levels of adrenaline in the plasma of patients with open surgery at the end of surgery were decreased (Figure 2F).

## 4 Discussion

In our study, 5 participants out of the total 9 participants with POD had POD on postoperative day 1, and 78% POD occurred within 2 days after the surgery. Similar to our findings, a previous study has shown that nearly 70% of patients with POD have the POD presentation within 2 days after spine surgery, and the number is about 85% within 3 days after surgery [27]. Surgery induces an acute systemic inflammation and neuroinflammation, which is often reduced a few days after surgery [9, 16, 28]. In addition, pain is the most severe within 2 to 3 days after surgery, and patients often receive opioids during this time [29]. Finally, patients must adapt to a new environment in the hospital after surgery. Neuroinflammation, pain, opioid use, and an unfamiliar environment in the phase of stress caused by surgery have been considered risk factors for POD [11, 13, 14, 15, 30] and may contribute to the phenomenon that POD is often seen in the early postoperative phase.

Patients with cardiac, vascular, and orthopedic surgeries often have a higher incidence of POD [11], possibly due to a higher potential of brain ischemia and micro‐thrombosis in these surgeries than in other surgeries. Surgical trauma induces neuroinflammation [9, 16], a neuropathological process that is considered important for POD [14, 15]. Our previous study has shown that the degree of surgical trauma is associated with the severity of neuroinflammation and the development of POCD in mice [9]. A major question is whether surgical trauma severity plays a role in the development of POD. There is conflicting evidence on whether minimal invasive surgery reduces POD [11, 31, 32]. Our study showed that POD occurred in patients with open surgery for their thoracic aortic aneurysm but not in patients with stent placement for the same disease. These results suggest that surgical trauma is an important factor in determining whether POD will occur. Of note, patients with open surgery had a much longer surgery/procedure time than patients with stent placement. However, surgical length was not different between patients with and without POD in patients with open surgery and was not identified as a risk factor for POD in these patients. The reasons for these findings may include that (1) surgical length may not be equal to surgical trauma severity and (2) surgical trauma needs to have other risk factor(s) to induce POD.

Our current data showed that patients with stent placement had a shorter duration of general anesthesia but the average length of anesthesia for this procedure was more than 4 h. One question is whether the difference in anesthesia length between patients with stent placement and open surgery contributes to the different finding in POD incidence between them. Our previous study has shown that exposure to general anesthesia for 6 h dose not induce neuroinflammation and POCD in mice [9]. Patients with neuraxial anesthesia or regional blocks and without general anesthesia for orthopedic surgeries also develop POD [12, 13]. Anesthetic choice (intravenous vs. volatile anesthetics) does not appear to affect the incidence of POD after surgery [11, 33]. Finally, anesthesia depth monitoring reduces the amount of anesthetics used and cumulative time with electroencephalogram waveform suppression [25]. However, conflicting findings have been reported on whether anesthesia depth monitoring reduces POD [25, 34]. These results suggest that general anesthetics, and the depth and duration of general anesthesia may not be the major contributors to the development of POD. Consistent with this possibility, patients with POD did not have longer anesthesia duration than those without POD in patients with open surgery. In this context, it may be interesting to note that general anesthetics can induce neuroprotection and inhibit detrimental stimulus‐induced neuroinflammation and damage of blood–brain barrier [35, 36, 37]. However, general anesthetics‐induced detrimental effects on the brain have also been reported in animal studies [36, 37]. These dual effects may explain why conflict findings on the general anesthetics for the development of POD have been reported.

Neuroinflammation is considered an important neuropathological process for both POCD and POD [14, 15, 28, 38]. As expected, IL‐1β, a proinflammatory cytokine [39], in the blood was increased in patients after open surgery whereas it was not changed in patients with stent placement. These results suggest that surgery but not stent placement induces systemic inflammatory responses. Surgery also increased the concentrations of NFL in the blood, indicating the injury of neurons [40]. However, the levels of IL‐1β and NFL in the CSF were not changed in patients with open surgery or stent placement. Surgery‐induced increase of proinflammatory cytokines and NFL in the CSF has been reported [41, 42, 43, 44]. The reason for failing to detect such an increase in our study is not clear but may be due to different surgical populations and time points for sample collection, and the use of CPB. Interestingly, the levels of adrenalin in the plasma at the end of surgery were decreased and the levels of corticosterone were not changed in patients with open surgery and stent placement. Adrenaline and corticosterone in the blood are often used as biomarkers for stress and are shown to increase in patients with surgery in many studies [45, 46]. However, no change [47] or decrease [48] during the perioperative period has also been reported. The reason for this inconsistent finding is not known but may be related to the use of different anesthetics and surgeries. Together, the biochemical results of our study indicate that open surgery but not stent placement induces systemic inflammation and neuronal injury, which is consistent with the finding that only patients with open surgery develop POD. Of note, surgery can increase the permeability of the blood–brain barrier, possibly due to systemic inflammation [49, 50]. This increased permeability may facilitate the transmission of systemic inflammation to the brain and the exchange of cells and molecules between blood and brain tissues in patients with surgery. These effects may play a role in the development of POD.

Our results showed that patients with open surgery had a higher ASA physical classification and used CPB more frequently than patients with stent placement. Since open surgery is associated with POD, poorer ASA physical status and the use of CPB may be risk factors for POD. However, patients with POD did not have a poorer ASA physical status and more frequent use of CPB than those without POD when patients with open surgery were considered. Multivariate analysis did not identify ASA physical status and CPB use as risk factors for POD. Thus, it is unclear whether poorer ASA physical status and the use of CPB are risk factors for POD among patients with open surgery for thoracic aortic aneurysm. However, poorer ASA physical status and the use of CPB are considered risk factors for POD [11, 51] and may be factors that facilitate the development of POD in patients with open surgery compared with patients with stent placement.

It was not surprising to see that more patients in the open surgery group used CPB and planned hypothermia and that patients with open surgery had a body temperature close to normal at the end of surgery than patients with stent placement, possibly due to the efficient rewarming by CPB. One novel finding from our study is that low body temperature at the end of surgery is a risk factor for POD. Patients with POD have a lower body temperature during cardiac surgery than patients without POD [52]. Inconsistent results have been reported about the role of low body temperature during non‐cardiac surgery in POD [53, 54]. A meta‐analysis does not find an association between intraoperative hypothermia and POD in non‐cardiac surgery [53]. Consistent with this finding, our study did not find a difference in the use of planned hypothermia during open surgery in patients with and without POD. Different from the previous studies that are retrospective reviews of patients' charts [52, 54], our study is a prospective observational study. Importantly, we have shown that low body temperature at the end of surgery is associated with POD. The role of body temperature at this time point in POD has not been reported. Low body temperature may induce various detrimental effects, such as enhancing stress response in awakening patients and decreasing brain activities [55, 56]. These effects may contribute to the development of POD. Hypothermia during surgery may not be associated with POD because patients are under general anesthesia that inhibits mild hypothermia‐induced stress. Thus, tissue trauma induced by surgery and low body temperature at the end of surgery may work together to facilitate the development of POD. Although patients with stent placement had a lower body temperature than patients with open surgery at the end of the procedure, stent placement may not induce significant tissue injury and, thus, does not cause POD.

As expected, patients with open surgery had a longer ICU and hospital stay than patients with stent placement. However, patients with POD did not have a longer ICU and hospital stay than patients without POD in the open surgery group. This no difference may be because patients after open surgical repair of thoracic aortic aneurysm have a long ICU and hospital stay and POD often has occurred within the ICU stay. Also, patients with POD were not older than those without POD. This no difference may be due to relatively young patients having open surgery in our study.

Our study has significant implications. Since low body temperature at the end of surgery may be associated with POD in patients with open surgery for thoracic aortic aneurysm, it may be better if those patients are warmed up well by CPB since the majority of those patients will be on CPB. Also, if our finding in thoracic aortic surgery is applicable to other surgery types, keeping patients warm will reduce POD, an additional benefit for maintaining the body temperature of patients in our practice. Finally, our study provides relatively good evidence for the role of surgical trauma in the development of POD. Practices that can reduce trauma may be used to reduce POD.

Our study has limitations. First, our case numbers are relatively small. However, these cases are very special to test our hypothesis that surgical trauma is a major contributor for POD. Also, no patient received steroids during the perioperative period, which eliminates the potential confounding effects of steroids on POD and inflammatory responses [57]. Second, our patient population has undergone thoracic aortic procedures. It is not known whether some patients have micro‐embolization in the brain, which then contributes to the brain dysfunction, such as POD.

## 5 Conclusions

In summary, we have shown that surgical trauma may be a major risk factor for POD. This surgical trauma may work with low body temperature at the end of surgery to facilitate the development of POD.

## Author Contributions

Z.Z. conceived the project; Z.Z. designed the studies; L.G.F., W.S., A.S.T., and S.Y. performed the experiments. L.G.F. and W.S. did the initial data analysis and drafted the methods section. Z.Z. performed the final analysis of the data and wrote the manuscript.

## Ethics Statement

The study protocol was approved by the University of Virginia Institutional Review Board (protocol number: 15903).

## Consent

All participants were consented for participation.

## Conflicts of Interest

The authors declare no conflicts of interest.

## Data Availability

The data that support the findings of this study are available from the corresponding author upon reasonable request.
